# Advanced Corneal Cross-Linking System with Fluorescence Dosimetry

**DOI:** 10.1155/2012/303459

**Published:** 2012-06-27

**Authors:** Marc D. Friedman, Radha Pertaub, David Usher, Evan Sherr, Pavel Kamaev, David Muller

**Affiliations:** Research and Development, Avedro Inc., Waltham, MA 02451, USA

## Abstract

*Purpose*. This paper describes an advanced system that combines corneal cross-linking with riboflavin with fluorescence dosimetry, the ability to measure riboflavin diffusion within the cornea both before and during UVA treatment. *Methods and Results*. A corneal cross-linking system utilizing a digital micromirror device (DMD) was assembled and used to measure diffusion coefficients of 0.1% riboflavin in 20% dextran in porcine eyes. A value of (3.3 ± 0.2) × 10^−7^ cm^2^/s was obtained for the stroma. Diffusion coefficients for the transepithelial formulation of 0.1% riboflavin in 0.44% saline and 0.02% BAK were also measured to be 4.7 ± 0.3 × 10^−8^ cm^2^/s for epithelium only and (4.6 ± 0.4) × 10^−7^ cm^2^/s for stroma only. Riboflavin consumption during a UVA treatment was also demonstrated. *Conclusion*. A new advanced corneal cross-linking system with fluorescence dosimetry of riboflavin has been demonstrated. It is hoped that this method may play a significant role in determining the underlying mechanisms of corneal cross-linking and assist with the development of additional riboflavin formulations. Moreover, dosimetry may prove valuable in providing a method to account for the biological differences between individuals, potentially informing cornea-specific UVA treatment doses in real time.

## 1. Introduction

Corneal cross-linking with riboflavin was first described by Spoerl et al. [1] and later used for the treatment of keratoconus [2–5]. It has become a widely used procedure. The standard corneal cross-linking Dresden Protocol is described as the instillation of 0.1% riboflavin solution in 20% dextran for 30 minutes with drops every 3–5 minutes, followed by 365 nm UVA illumination with 3 mW/cm^2^ for 30 minutes for a total dose of 5.4 J/cm^2^ with continued instillation of riboflavin drops every 5 minutes [2]. Recently, several new protocols using different riboflavin formulations (transepithelial, nondextran, and higher concentration riboflavin) and different UVA illumination irradiance levels (accelerated cross-linking) are being developed and used clinically.

In addition several new applications have emerged. These include post-LASIK regression minimization for hyperopia [6], treatment of infectious keratitis [7, 8], corneal melts [9], Acanthamoeba Cysts [10–12], and postthermal keratoplasty stabilization [13]. 

Dosimetry is the ability to quantitatively measure the concentration of riboflavin penetrating the cornea to a given depth as well as the amount of riboflavin being consumed during UVA illumination. As more protocols, formulations and uses for corneal cross-linking with riboflavin are developed, cross-linking systems that use dosimetry could be of significant importance. Real-time dosimetry may be used in determining the underlying mechanisms of corneal cross-linking and assist with the development of additional riboflavin formulations. It may also be used clinically to account for the biological differences between individuals, potentially informing cornea-specific UVA treatment doses with real-time feedback. 

The history of dosimetry in phototherapy has concerned mostly bulk nontransparent tissues. Wilson et al. [14] described explicit dosimetry as the average measure of photosensitizer concentration in or around the target tissue just prior to light treatment, and the light fluence at some points within or around the target volume. He defined implicit dosimetry as the use of photosensitizer photobleaching to generate the dose metric. The cornea is unique in that it is optically transparent making it well suited for both explicit and implicit dosimetry through fluorescence detection of corneal cross-linking with riboflavin as well as riboflavin depletion by UVA. 

Fluorescence detection through the use of fluorescein in ophthalmology is widely accepted as a method for determining corneal epithelial permeability. Kruijf et al.[15] described a simple method utilizing the Fluorotron Master (Coherent Radiation Inc., Palo Alto, CA, USA) fitted with a special lens (anterior segment adaptor) for detailed scanning of the anterior segment of the eye to determine corneal epithelial permeability. Joshi et al. [16], almost a decade later, used the Fluorotron Master to calculate epithelial permeability. Because of insufficient resolution, concentrations could not be estimated, and instead the total masses in the tear film and in the cornea derived from the area under the profile were used to calculate the epithelial permeability.

More recently, Søndergaard et al. [17] used confocal fluorescence microscopy to evaluate the distribution of riboflavin in the corneal stroma under varying concentrations and application time. Cui et al. [18] used an 800-nm femtosecond laser to perform two-photon fluorescence (TPF) axial scanning to measure the concentration of fluorescein and riboflavin across the central corneal depth in a freshly enucleated feline globe model. 

This paper describes an advanced system that combines both corneal cross-linking with riboflavin and fluorescence dosimetry. A digital micromirror device (DMD) UVA projector and imaging setup was assembled and used to measure riboflavin diffusion coefficients of both epithelium and stroma and to measure riboflavin consumption during a UVA treatment. Future development of this system and its implications are discussed. 

## 2. Material and Methods

### 2.1. Fluorescence Dosimetry Experiments Using a Bench Top Digital Micromirror Device [DMD] with UV Source

**Figure 1:**
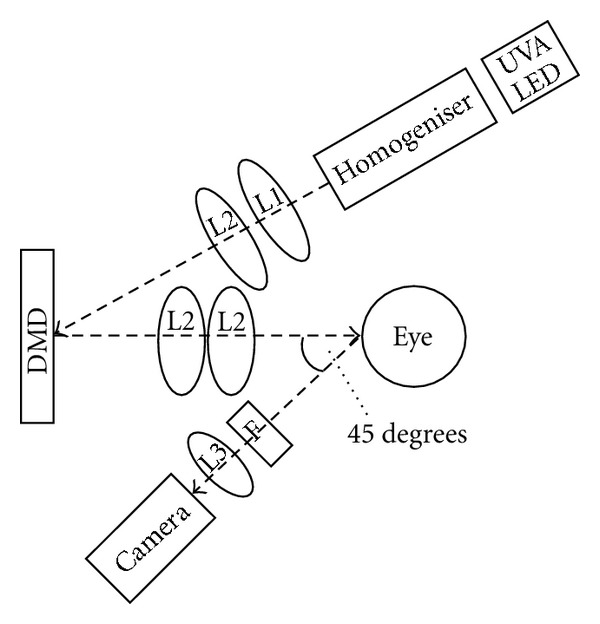
Block diagram of bench top DMD system configuration.

A bench top corneal cross-linking system using a DMD with an additional fluorescent dosimetry camera was developed. The DMD is the same technology utilized in digital projectors and has the advantage of being able to create any unique beam shape or intensity profile. The bench top setup seen in Figure 1 is comprised of an UVA LED illuminator (UV LED NCSU033B(T), Nichia Co., Japan) with a wavelength of 365 nm. Light emitted from the illuminator (beam uniformity of ±3% Root Mean Square) is directed onto a digital micro-mirror device (DMD) (Texas Instruments, Model XGA UV DMD) via a homogenizer and lenses L1 (NT49-695, Edmund Optics) and L2 (NT48-285, Edmund Optics). Light from the surface of the DMD is imaged with the L2 doublet and centered onto the eye coincident with its optical axis. A 1.3 mega pixel camera (Basler acA1300-30 gm), with L3 (Computar MLM3X-MP) and a long-pass filter, F, with a cut-off wavelength 420 nm (LP415 UV Dichroic Blocking Filter, Midwest Optical Systems), is mounted and centered at a 45° angle to the apex of the eye and its cornea.

**Figure 2:**
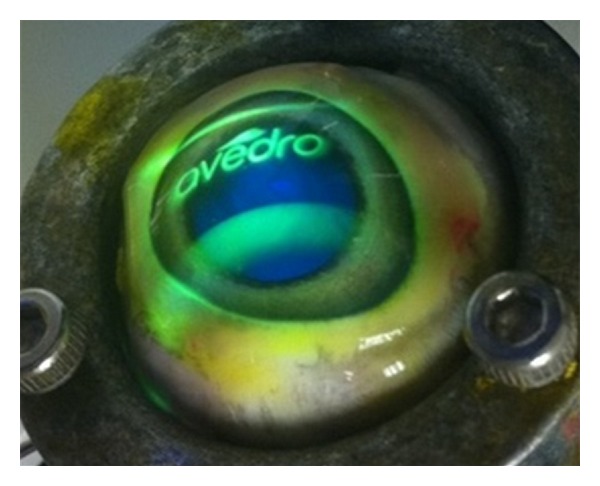
Example of the flexibility of UV source patterns projected on an excised porcine eye.

The DMD can be configured to project UVA illumination of any arbitrary pattern with 256 (8-bit) levels of intensity which is controlled by a video signal that can be updated at a rate of 60 Hz. This allows for rapid switching between an illumination profile intended for cross-linking treatment and that of a profile intended for fluorescent dosimetry. The system can, therefore, rapidly switch modes such that dosimetry measurements can be taken at intervals prior to and during the treatment. This is illustrated in Figure 2 where an image corresponding to a projected pattern by the DMD bench top system is shown. 

**Figure 3:**
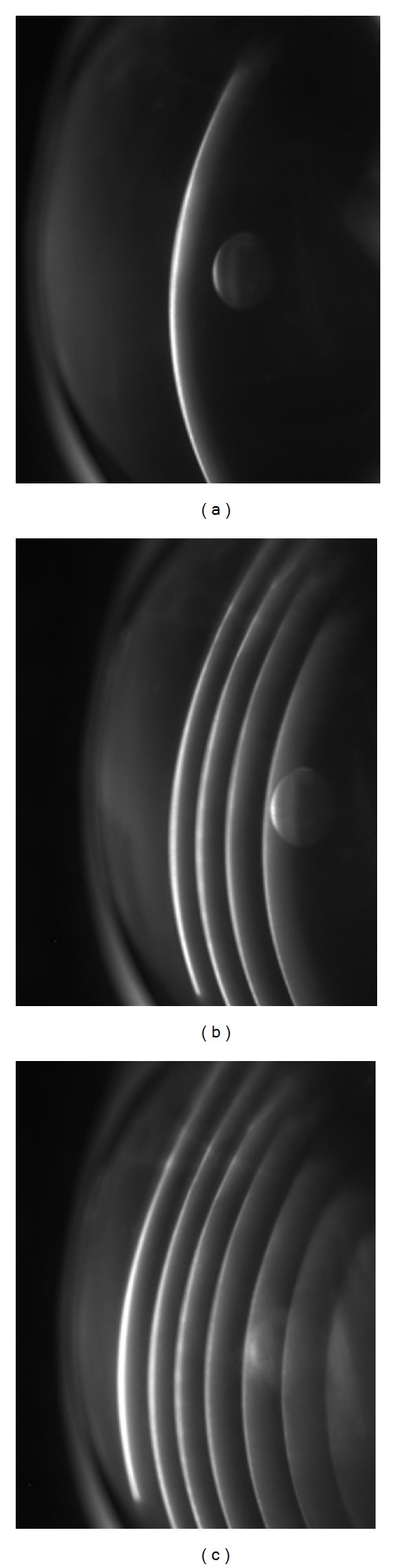
Example images of a fluorescing porcine cornea. Three images of projected patterns by the DMD bench top system (a) a vertical slit, (b) 4 parallel vertical slits, and (c) 7 parallel vertical slits.

During fluorescent dosimetry measurement, Figure 3, one or more vertical lines are projected. The first image shows one vertical line image, the second shows four vertical line images, and the third shows seven vertical line images. Each line image shows the cross-sectional distribution of riboflavin through the cornea at its spatial location. Riboflavin distribution within the cornea can be instantly measured at various points by projecting multiple line images onto the cornea. Additionally, this method may be used to provide corneal topography of the corneal surface as well as the riboflavin distribution through the cornea since the leading edge of the fluorescent image is a measure of the cornea's curvature. 

In the following experiments, the DMD was configured to project a vertical one-pixel line width (approximately 25 *μ*m) of illumination onto the corneal surface and set to an output irradiance of 30 mW/cm^2^. 

Three separate experiments were performed. The first experiment was to measure riboflavin diffusion through the cornea without epithelium, the second experiment was to measure riboflavin diffusion through the cornea with epithelium, and the third experiment was to measure riboflavin consumption during a UVA treatment. 

In experiment 1 porcine eyes (*n* = 3) were supplied 1 day after mortem by Sioux-Preme (Sioux Center, IA, USA) and pressurized to approximately 15 mmHg with a saline column approximately 20 cm above the eye. Epithelial debridement was performed with a flat blade. An image was taken prior to the application of riboflavin to serve as a baseline image. A solution of 0.1% riboflavin in 20% dextran was instilled onto the corneas every 30 seconds for 3 minutes. The corneas were then rinsed with buffered saline, and an image was recorded. This procedure was then repeated with images recorded every 3 minutes for a total of 21 minutes. In the second experiment, the previous experimental protocol was repeated this time using porcine eyes (*n* = 3) with their epithelium intact and a transepithelial solution of 0.25% riboflavin in 0.44% saline with 0.02% benzalkonium chloride (BAK). 

In the third experiment, several drops of 0.25% riboflavin in 44% saline were instilled to cover deepithelialized eyes (*n* = 6) every 30 seconds for a total of 10 minutes. The eyes were rinsed with saline. Images were then recorded for each of the eyes as a baseline image. UVA treatment of eyes (*n* = 3) was placed under a uniform 10 mm diameter UVA beam with a wavelength of 365 nm at an irradiance of 30 mW/cm^2^. After one minute the UVA treatment was temporarily stopped, and fluorescence dosimetry images were taken. This was repeated 5 more times to obtain images up to a total UVA dose of 10.8 J/cm^2^. A set of control eyes (*n* = 3) underwent the same procedure but without UVA irradiation. Particular care was taken to keep the intervals between UVA irradiation and imaging constant.

### 2.2. Franz Cell Experiments

Porcine eyes were supplied 1 day after mortem by Sioux-Preme (Sioux Center, IA, USA) and were warmed to room temperature and allowed to fully swell. An automated Hansatome microkeratome (model HT 230, Chiron Vision, Hansa Research & Development, Inc., Miami, FL) was used to create corneal flaps by excision. The average thickness of the flaps was approximately 100 *μ*m as measured with an ultrasonic pachymeter (DGH-550 Pachette 2, DGH Technology, Exton, PA, USA).

The diffusivity of riboflavin through cornea was measured by experimentation using a temperature controlled (37°C) Franz cell (PermeGear, Inc. Hellertown, PA, USA). Corneal flaps were placed between the receiver and donor compartments of the Franz cells. Receiver compartments were filled with saline, and donor compartments were filled with a riboflavin solution for each formulation tested. Corneal flaps (*n* = 3) for each formulation were tested. The amount of riboflavin passing through the corneal flaps per time was measured by means of spectrophotometry, and diffusivity of riboflavin was calculated using Fick's first law. 

### 2.3. Image Analysis

Images were exported and postanalyzed using ImageJ software (http://rsbweb.nih.gov/ij/). Cross-sectional intensity profiles were plotted for each image for both before and after the addition of riboflavin. Control images of the cornea before instillation of riboflavin provided baseline intensities that were subtracted from the intensities of the cornea with riboflavin. 

A cosine correction factor was used to adjust for the 45° angle of incidence in order to obtain the correct corneal depth measurement. Calibration tests performed measured a resolution of 12.2 *μ*m per pixel. The resulting intensity profiles of riboflavin in the corneas were then fitted using the solution to Fick's second law of diffusion equation

(1)C=C0(1−
erf
(x2Dt)),

where *C* is concentration in the medium diffused, *C*_0_ is initial concentration at boundary, *x* is spatial distance, *D* is diffusion coefficient, and *t* is the time needed for diffusion.

Since riboflavin fluoresces linearly with concentration, (1) becomes as follows:

(2)I=I0(1−
erf
(x2Dt)),

where *I* is intensity in the cross-section of the cornea, and *I*_0_ is initial intensity at the boundary.

## 3. Results 

### 3.1. DMD Bench Top Experiments

**Figure 4:**
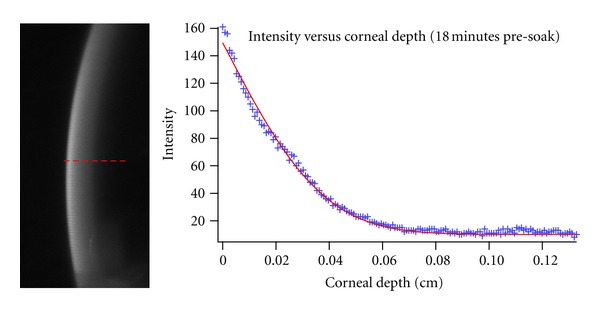
The image on the left shows the intensity profile after 18 minutes of presoak with 0.1% riboflavin in 20% dextran. The corresponding plot on the right shows the intensity versus the corneal depth (blue marker) and the curve fit to the experimental data (red line).

For the experiment with riboflavin in dextran and no epithelium, a representative analysis can be seen in Figure 4. The raw image of the slit projection can be seen together with the extracted intensity profile. The corresponding model fit using (2) is also shown. An average diffusion coefficient of (3.3 ± 0.2) × 10^−7^ cm^2^/s was recorded for this formulation of riboflavin. This average was calculated using all images recorded for soak times from 6 minutes through 21 minutes for all three eyes. This value compares well with the value of (3.8 ± 0.8) × 10^−7^ cm^2^/s obtained using the Franz cell approach.

**Figure 5:**
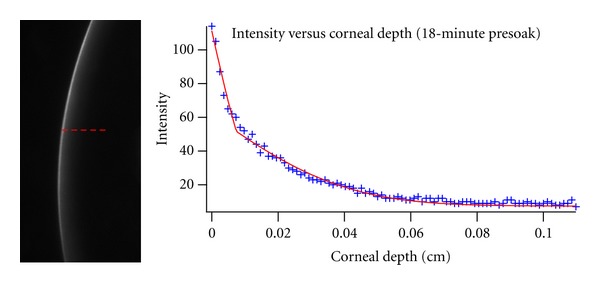
The image on the left shows the intensity profile after 18 minutes of presoak with 0.1% riboflavin in 0.44% saline, 0.02% BAK. The corresponding plot on the right shows the intensity versus the corneal depth (blue marker) and the biphasic curve fit to the experimental data (red line).

For the experiment with riboflavin in BAK solution and epithelium intact, a representative analysis can be seen in Figure 5. The analysis of the average value of the diffusion coefficient was performed for soak times 6 minutes through 21 minutes for all three eyes. In this experiment, the riboflavin diffuses through both the epithelium and the stroma, each having its own diffusion coefficient. Therefore, the curve fit was done in two parts: coefficients *C*_1_ and *D*_1_ for boundary intensities at the top of the epithelium and a diffusion coefficient in epithelium alone (for *x* between 0 and 75 *μ*m) and coefficients *C*_2_ and *D*_2_ for boundary intensity at the top of the stroma and a diffusion coefficient in stroma alone, respectively (for *x* > 75 *μ*m). 

**Figure 6:**
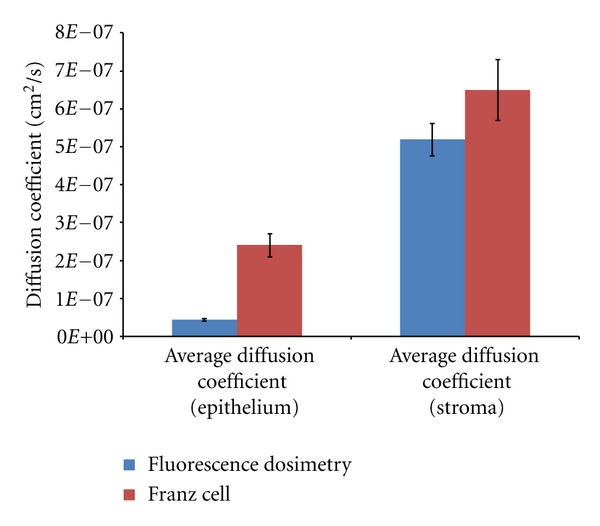
Comparison between diffusion coefficients obtained with fluorescence imaging versus a Franz cell for a 0.1% riboflavin in 0.44% saline, 0.02% BAK formulation with and without epithelium.

A diffusion coefficient of (4.7 ± 0.3) × 10^−8^ cm^2^/s was obtained for the epithelium only and a diffusion coefficient of (4.6 ± 0.4) × 10^−7^ cm^2^/s for the stroma only for the fluorescent imaging. These values are slightly lower than the values obtained using the Franz cell method, (2.4 ± 0.3) × 10^−7^ cm^2^/s for the epithelium and stroma combined and (6.5 ± 0.8) × 10^−7^ cm^2^/s stroma only (Figure 6). 

**Figure 7:**
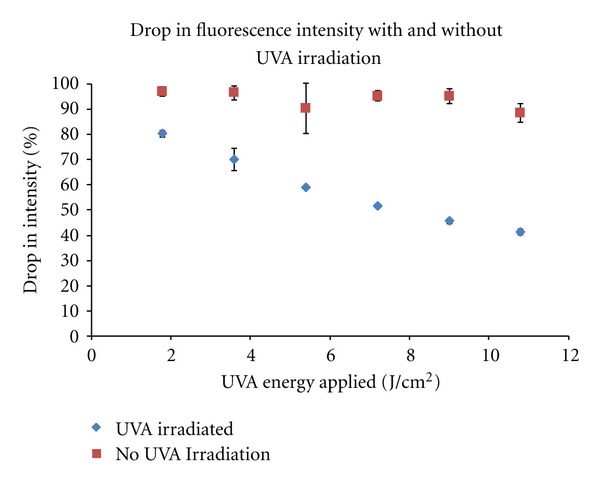
Drop in fluorescence intensity of the top 12 microns of the cornea with and without UVA dose.

For the third experiment, the percentage drop in fluorescence intensity detected in the top 12 microns of the cornea is seen in Figure 7 with respect to the controls.

## 4. Discussion

The fluorescence dosimetry experiment using the bench top DMD with UVA source demonstrated that the value of diffusivity without epithelium compared well with those obtained with the Franz cell experiment. For the transepithelial formulation in the second experiment, the values obtained using the Franz cell for the diffusion of riboflavin with epithelium are an effective diffusion coefficient of combined epithelium and stroma. The Franz cell method is not able to deconvolve the two diffusion coefficients separately where fluorescence imaging can. The clinical implication of this finding is that fluorescence dosimetry may also be able to differentiate and account for variable epithelial hypoplasia and hyperplasia across the cornea, a very common condition in keratoconus.

The third experiment demonstrates that photo-dissociation of riboflavin over time can be detected and that, without the addition of riboflavin, the riboflavin slowly continues to diffuse into the cornea.

The current work assumes no scattering. This is an approximation as there is light scattering in the cornea at 370 nm. Second-order enhancements are planned which will incorporate light scattering by the cornea into our image analysis models. This modeling will also account for the UVA absorbed as well as the fluorescence produced to determine the dose delivered and how this equates to the amount of corneal cross-linking produced. Temperature effects, increased depth of focus, higher resolution, calibration methods, automated alignment including automatic *x*, *y*, and *z* fine alignment, cyclotorsion compensation based on eye, and landmark image analysis are all being investigated in an effort to produce a system with more consistent results.

This advanced system has several unique features which lend itself to possible real-time measurement in a combination UVA treatment and fluorescence-monitoring system. Beam uniformity and irradiance calibration at the corneal plane are simplified utilizing a DMD since each individual micromirrors average output power is adjustable. The system may also be used for customized corneal cross-linking procedures where the DMD patterning and pretreatment plan is based on corneal topography and or optical coherence tomography. Variables such as corneal elevations, power maps, *k* readings, corneal thickness maps, and epithelial thickness maps may all be utilized to create an optimized cross-linking pretreatment plan and procedure. Finally, during UV treatment, this treatment plan may also respond and compensate to live dosimetry readings as riboflavin is consumed and measured at various depths. We are in the process of investigating these possibilities.

## 5. Conclusion

A new advanced corneal cross-linking system with fluorescence dosimetry with riboflavin has been demonstrated. It is hoped that this method may someday play a significant role in determining the underlying mechanisms of corneal cross-linking and assist with the development of additional riboflavin formulations. Moreover, dosimetry may prove valuable in providing a method to account for the biological differences between individuals, potentially informing cornea specific UVA treatment doses in real time.
